# PCRRT-ICONIC critical care pediatric nephrology course: the global prevalence of COVID-19 and associated sequelae

**DOI:** 10.3389/fneph.2022.1008629

**Published:** 2022-10-05

**Authors:** Rupesh Raina, Nina Vijayvargiya, Riti Kalra, Hui-Kim Yap, Nikhil Nair, Khalid Alhasan, Giovanni Montini, Aarushi Narang, Mignon McCulloch, Melvin Bonilla-Felix, Arvind Bagga, Quen Mok, Marcelo de Sousa Tavares, Vera Koch, Franz Schaefer, Cavagnaro Felipe, Tim Bunchman, Sidharth Sethi

**Affiliations:** ^1^ Akron Nephrology Associates, Department of Nephrology, Cleveland Clinic Akron General Medical Center, Akron, OH, United States; ^2^ Department of Nephrology, Akron Children’s Hospital, Akron, OH, United States; ^3^ Department of Pediatrics, Yong Loo Lin School of Medicine, National University of Singapore, Singapore, Singapore; ^4^ Khoo Teck Puat-National University Children’s Medical Institute, National University Hospital, Singapore, Singapore; ^5^ Department of Internal Medicine, Case Western Reserve University School of Medicine, Cleveland, OH, United States; ^6^ Pediatric Department, College of Medicine, King Saud University, Riyadh, Saudi Arabia; ^7^ Department of Paediatrics, Yong Loo Lin School of Medicine, National University of Singapore, Singapore, Singapore; ^8^ Pediatric Renal and Solid Organ Transplant Unit, Red Cross War Memorial Children's Hospital, University of Cape Town, Cape Town, South Africa; ^9^ Department of Pediatrics, University of Puerto Rico, San Juan, Puerto Rico; ^10^ Department of Pediatrics, Division of Nephrology, All India Institute of Medical Sciences, New Delhi, India; ^11^ Department of Paediatric Critical Care, UCL Great Ormond Street Institute of Child Health, London, United Kingdom; ^12^ Pediatric Nephrology Unit, Nephrology Center, Santa Casa de Belo Horizonte Hospital, Belo Horizonte, Minas Gerais, Brazil; ^13^ Department of Pediatrics, University of Sao Paulo Medical School, Pediatric Nephrology Unit Instituto da Criança, Hospital das Clinicas University of Sao Paulo Medical School, Sao Paulo, Brazil; ^14^ Division of Pediatric Nephrology, Center for Pediatrics and Adolescent Medicine, Heidelberg, Germany; ^15^ Department of Pediatrics, Clínica Alemana de Santiago, Facultad de Medicina Clínica Alemana-Universidad del Desarrollo, Santiago, Chile; ^16^ Pediatric Nephrology & Transplantation, Children’s Hospital of Richmond at Virginia Commonwealth University, Richmond, VA, United States; ^17^ Pediatric Nephrology & Pediatric Kidney Transplantation, Kidney and Urology Institute, Medanta, The Medicity Hospital, Gurgaon, India; ^18^ Fondazione IRCCS Ca' Granda Ospedale Maggiore Policlinico, Nephrology, Dialysis and Pediatric Transplant Unit, Milan, Italy; ^19^ Department of Clinical Sciences and Community Health, University of Milan, Milan, Italy

**Keywords:** COVID-19, pediatrics, nephrology, acute kidney injury, multisystem inflammatory syndrome, chronic kidney disease

## Abstract

After nearly three years of the COVID-19 pandemic, research has affirmed that COVID-19 is more than just a respiratory virus. There have been significant breakthroughs made surrounding the development of acute kidney injury (AKI) and chronic kidney disease (CKD), in pediatric populations. Additionally, patient populations susceptible to renal complications consist of pediatric transplant recipients, multisystem inflammatory syndrome (MIS-C), and dialysis. Although research is gradually becoming more available surrounding this prevalent topic, knowledge is sparse on the deleterious effects of COVID-19 on pediatric patients with kidney disease and requires more in-depth analysis. The virtual international conference, Pediatric Critical Care Nephrology & Dialysis Course, on August 7th, 2021, reviewed the severe cases of COVID-19 in the global pediatric population. By integrating international perspectives, statistics, techniques, and treatments for managing renal complications, we further develop scientific understanding of the renal complications seen in children with COVID-19 globally.

## Introduction

In December of 2019, Wuhan Municipal Health Commission reported pneumonia cases of unknown origin, which were soon recognized as SARS-CoV-2, COVID-19. In March of 2020, COVID-19 became a global pandemic.

Symptoms of COVID-19 included fever, cough, and shortness of breath. Additional identifying symptoms included loss of sense of smell and taste, headache, fatigue, nausea, and diarrhea. Many patients were asymptomatic, contributing to the rapid spread of the disease. The risk of COVID-19 increases with age as older individuals with underlying health conditions may require hospitalization or intensive treatment. Conversely, according to the Centers for Disease Control and Prevention’s (CDC) U.S. COVID-19 report as of April 2020, out of 149,082 cases, only 2,572 cases occurred in patients <18 years (1). A study by Dong et al. reported that of the 94.1% observed COVID-19 pediatric patients, 4.4% were asymptomatic, 50.0% had a mild course (those who usually recover at home with ample care and isolation), and 38.8% had moderate (those who must be monitored closely and sometimes hospitalized) symptoms, supporting that clinical manifestation of COVID-19 in the pediatric population is milder, in comparison to adults (2). This was supported by Dr. Mignon McCulloch, the South African representative from the University of Cape Town, who reported only five pediatric deaths due to COVID-19 (Delta variant) in 18 months in South Africa. Nevertheless, severe complications, including the multisystem inflammatory syndrome (MIS-C), are still prevalent in the pediatric population and further observation of the short and long-term prognosis is important.

Although COVID-19 is a respiratory virus, ample research has suggested that pediatric patients with kidney disease are at severe risk for deleterious outcomes. The nephrology community has reported an increased prevalence of several kidney complications in pediatrics such as acute kidney injury (AKI) and chronic kidney disease (CKD). Patient populations including pediatric transplant recipients, multisystem inflammatory syndrome (MIS-C), and dialysis are especially susceptible.

The virtual international conference, Pediatric Critical Care Nephrology & Dialysis Course, on August 7th, 2021, reviewed these severe cases of COVID-19 in the pediatric population, focusing on kidney complications in children with COVID-19. This conference emphasizes that while COVID-19 is primarily a respiratory illness, other organs are also at risk. This conference hosted representatives across the globe, which offered international perspectives on the pandemic in various countries.

## Acute kidney injury in COVID-19 in pediatric patients

AKI is a known complication of COVID-19 and is considered an adverse prognostic factor for survival. While the severe disease is often seen in adult COVID-19 patients, complications are prevalent among pediatric populations as well. Dr. Rupesh Raina, the United States representative, describes the AKI incidence rate of pediatric COVID-19 patients at 26.60% whereas only 15.60% in adult COVID-19 patients. However, the mortality rate and use of kidney replacement therapy (KRT) are significantly lower in children than in adults. Dr. Raina reports that less severe outcomes in children are due to a more active innate immune response, fewer comorbidities, different maturation distribution, viral receptor function, and different RAS receptors which respond to inflammatory pathogens. COVID+ children with AKI have a significantly higher risk of mortality, length of hospital stay, and greater use of kidney support therapy than COVID+ children without AKI. Dr. Arvind Bagga extended Dr. Raina’s analyses to Indian children, as he reported a 22.8% incidence rate of AKI in infected children with COVID-19 at the Tertiary care hospital in Delhi, of which more than 33% progressed to Stage 3, with a mortality rate of 26%. Additionally, Dr. Khalid Alhasan, the Saudi Arabia representative, described that ⅕ of pediatric COVID+ hospital patients developed AKI, of which ⅓ were admitted to the PICU, and also describes an increase in the incidence of AKI over time, due to the prevalence rising and more data being collected. Together, Dr. Raina, Dr. Bagga, and Dr. Alhasan emphasized that complications in COVID-19 often extend beyond respiratory distress to renal disorders; it is vital to share this knowledge as we continue to tackle the morbidity and mortality associated with this virus. **Table 1** addresses AKI incidence rate by country.

**Table 1:** AKI Incidence Rate by Country.

Country	AKI Incidence Rate - COVID-19 Population	Source
United States of America	26.60%	Conference
India	22.8%	Conference
Saudi Arabia	20%	Conference
Latin America	60%	Conference
Italy	1.2%	Livingston and Bucher et al. (3)
England	29%	Stewart et al. (4)
China	2.7%	Dong et al. (2)
France	52%	Toubiana et al. (5)

## Chronic kidney disease in COVID-19 pediatric patients; what we know

The rise of COVID-19 cases has exacerbated the prevalence of CKD. Those who recover from COVID-19 and experience AKI are at increased risk of developing CKD. Dr. Bagga alluded to the incidence of COVID-19 in children with CKD: Dr. Bagga introduced a retrospective study analyzing 88 male pediatric patients with COVID-19. In patients who had CKD, the proportion of patients developing AKI with CKD was 34%. There was an additional need for kidney replacement therapy in 11% of patients with CKD, nephrotic syndrome, and kidney transplant recipients. Among the study population, 12 CKD patients had stage 2 AKI, 12 patients had stage 3 AKI, 11 had respiratory failure, 4 developed shock, 1 had encephalopathy, and 3 died. Hence, although research does not yet point to a causal relationship between COVID-19 and CKD, diminishing kidney function in COVID-19 patients has been reported.

## Multisystem inflammatory syndrome

An analysis described by Dr. Raina reported that 74.29% of pediatric COVID-19 patients developed MIS-C. Dr. McCulloch described how to identify, diagnose, and treat MIS-C in children. 0-19-year-old individuals must present with a fever for three or more days, have higher inflammatory markers, present no other source of inflammation, have been exposed to or positive for COVID-19, and as seen in **Table 2**, and have two of the following:

**Table 2:** MIS-C in COVID-19.

1. Bilateral non-purulent conjunctivitis, rash, or signs of mucocutaneous inflammation 2. Shock or hypotension 3. Pericarditis, myocardial dysfunction, coronary irregularities measured by higher troponin/NT-proBNP or ECHO results, and valvulitis 4. Coagulopathy was identified by irregular PT and PTT and higher d-Dimers 5. Acute gastrointestinal issues including abdominal pain, vomiting, or diarrhea

In suspected MIS-C, it is essential to observe cardiac function by measuring heart rate and blood pressure. Kidney function observation is also encouraged by measuring fluid status and urine output. If necessary, a referral to a cardiologist, rheumatologist, nephrologist, or intensivist may be needed. It is also essential to treat organ damage through ventilation, inotropes, oxygen, and diuretics and treat pain when present. For specific management, patients should be given the option to enroll in international trials depending on location, including the SA-PED RECOVERY trial, studying the use of drugs such as aspirin, vitamin D, Intravenous Immune Globulin (IVIG), and corticosteroids. A response to treatment is observed by decreased C-reactive protein (CRP), normalization of cardiac function and heart rate, and apyrexial state. In addition to standard follow-up procedures, visits to cardiology rheumatology are needed following the discontinuation of prednisone and aspirin.

## The clinical course of COVID-19 in immunosuppressive pediatric patients with renal conditions

The conference’s German representative, Dr. Franz Schaefer, offered insight from the University of Heidelberg as he described the clinical course of COVID-19 in children with renal diseases receiving immunosuppressive therapy. Immunosuppressive adult patients experienced a more severe course of COVID-19 infection. A systematic review by Belsky et al. showed that adults with cancer and stem cell transplantation had a 20% mortality rate in comparison to the 10% mortality rate in the general population (6). On the other hand, pediatric cancer patients had similar mortality rates to the general population, prompting investigations into the clinical course of COVID-19 in immunosuppressive children.

Dr. Schaefer describes the 2020 global survey which studied immunosuppressive therapy for renal conditions in 113 COVID-19 patients aged 0-20 years. As seen in **Table 3**, among the population studied, the following underlying conditions were presented: kidney transplantation (46%), nephrotic syndrome (26%), Systemic lupus erythematosus (SLE) (10%), Anti-neutrophil cytoplasmic autoantibody (ANCA) vasculitis (2%), IgA nephropathy (IgAN) (2%), immunoglobulin A vasculitis nephritis-Henoch-Schönlein purpura nephritis (IgAVN-HSPN) (2%), atypical hemolytic uremic syndrome (aHUS) (2%), other glomerulonephritis (GN) (6%), and other (4%). 76% of patients were on glucocorticoids, 51% were on Tacrolimus (Tacro)/Cyclosporin (CsA), 54% were on Mycophenolate Mofetil (MMF)/Azathioprine (AZA), 7% were on mTOR inhibitors such as Rapamycin, 7% were on cyclophosphamide, 10% were on Rituximab, and 6% were on other monoclonal antibodies (MAB). This study identified no significant differences between kidney conditions and the severity of the virus’ clinical course. Additionally, no significant difference was identified in the virus’ clinical course in pediatric patients with renal conditions on immunosuppressive medications and the control group (healthy pediatric individuals). Even patients on Rituximab, who are B-cell depleted and hence cannot produce antibodies, exhibited a similar clinical course to the control group.

**Table 3:** 2020 Global Survey, Dr. Schaefer.

Sample Population	n = 113 COVID-19 patients, 0-20 years
Kidney transplantation	46%
Nephrotic syndrome	26%
SLE	10%
ANCA Vasculitis	2%
IgA nephropathy	2%
aHUS	2%
Other glomerulonephritis	6%
Other complications	4%

Dr. Schaefer describes another global survey that studied the use of Rituximab during the pandemic. The lead investigator gathered information from 82 nephrologists in 32 countries and reported a 12% reduction in the use of Rituximab during the pandemic due to unavailability or lack of necessity. This study reported a 7% infection rate for children who received Rituximab, which is similar to the infection rate in the general pediatric population, and showed that fewer Rituximab patients were admitted to the hospital than healthy controls. These findings also suggested that the use of Rituximab in pediatric patients does not differ from the general pediatric population, and further suggested that Rituximab does not increase the risk of COVID-19 infection in pediatric patients. Hence, Dr. Schaefer suggested that children on immunosuppressive therapy should follow the same prophylactic measures of masking and social distancing as the general population, suggesting that there is no current reason to isolate children on immunosuppressive therapy more than their healthy counterparts.

## Vaccine safety and efficacy in renal transplant patients

Concerns about the safety and efficacy of the COVID-19 vaccine in patients with or seeking kidney transplants were addressed at the conference. Findings described by Dr. Schaefer revealed an essential discrepancy between adult solid organ transplant recipients and the general population. This 2021 study by Kamar et al. showed decreased efficacy of the mRNA COVID-19 BioNTech-Pfizer vaccine in adult solid organ transplant recipients, as around 60% of the transplant patients did not have sufficient antibody levels. Of the studied patients, 87% were on glucocorticoids, 63% were on mycophenolate mofetil (MMF), 79% were on calcineurin inhibitor drugs (CNI), 30% were on mTOR (a class of drugs that inhibit the mechanistic target of rapamycin), and 12% were on belatacept. Administration of a third dose two months after the second dose provided most transplant patients with sufficient antibodies, suggesting that adult immunosuppressive patients might need a third dose to be sufficiently protected from the virus. Additionally, Dr. Giovanni Montini, who represented Italy at the conference, raised concerns about administering the COVID-19 vaccine to patients needing a kidney transplant due to concerns about contributing to transplant rejection. Dr. Schaefer and Dr. Montini addressed concerns regarding the safety and efficacy of the vaccine in renal transplant patients. These concerns may pose serious health problems for transplant recipients, as Dr. Bagga reported they often have a severe course of the disease: 11.6% died, 14.5% were hospitalized, 47% were admitted to the ICU, and 97% required ventilation. Hence, these concerns must be addressed through future research on vaccine administration in renal transplantation patients.

## COVID-19 in Britain

Representing the UK, Dr. Quen Mok, clinical specialist in pediatric and neonatal intensive care, described the preventative planning process installed to protect individuals from COVID-19. In March 2020, the UK responded to the COVID-19 crisis in Italy by increasing bed capacity, preparing new ICUs for COVID-positive patients, and changing medical rotations as necessary. Younger staff was elected to look after COVID ICU patients, and older staff were reassigned to less risky duties. While COVID-19 cases comprised just 1-5% of the pediatric population, Dr. Mok mentioned the challenges that children with COVID-19 faced, including restricted family visitation, fear of staff members in PPE, and lack of communication. Creative solutions involved making PPE child-friendly, video calls with families, and clear introductions. The speaker stressed the importance of early planning to confine positive and suspected cases, ensuring adequate PPE and training, and remaining knowledgeable on the current literature.

The mortality rate in COVID-19 pediatric patients was low, and the children exhibited a delayed onset of multisystem inflammatory response due to COVID-19. Pediatric patients requiring critical care were most often less than 1-month-old (these patients were born to COVID positive mothers), aged 10-14 years, of African ethnicity or Asian ethnicity, had a premature birth, had respiratory or cardiac comorbidity or were obese. COVID-19 treatment plans at GOSH included prophylactic anticoagulation, inotropic support, mechanical ventilation, and ECMO. In addition, Dr. Mok reported data on multi-organ system involvement in COVID-19 infection: patients had renal (91%), nervous system (50%) *and* gastrointestinal (98%) involvement, prothrombotic states (87%), and echo abnormalities and increased troponin and pro-b-type natriuretic peptide NT-proBNP (33%). Multi-organ system involvement in COVID-19 infection is recorded in **Table 4**. Hence, Dr. Mok suggested that, although pediatric patients constitute a smaller percentage of infected and critically ill patients, they too often suffer from complications in many organs.

**Table 4:** Multi-organ system involvement in COVID-19 infection, Britain.

Renal Involvement	91%
Neuronal Complications	50%
Gastrointestinal Involvement	98%
Prothromtic States	87%
Echo abnormalities/Increased troponin/NT-proBNP	33%

## COVID-19 in Latin America

Dr. Melvin Bonilla-Felix, president of the Latin American Pediatric Nephrology Association, a branch of the International Pediatric Nephrology Association (IPNA), conducted a Latin American survey about kidney complications in pediatric COVID-19 patients. In Latin America, poverty, poor sanitation, limited access to water, and lack of trust in the government were key factors that contributed to outbreaks. 60% of pediatric COVID-19 patients with no prior renal conditions developed AKI, of which 38% required kidney replacement therapy. Among pediatric patients with pre-existing renal conditions (nephrotic syndrome, dialysis, transplant, lupus nephritis, and tubulopathies) who contracted COVID-19, 33% did not require hospitalization, 44% required critical care, and 27% died. Dr. Bonilla-Felix discusses COVID-19 in pediatric patients across Latin America, which Dr. Marcelo de Sousa Tavares and Dr. Vera Koch expanded on as they discussed the pediatric pandemic experience specifically in Brazil.

Dr. Tavares and Dr. Koch described COVID-19 in the pediatric population in Brazil. Brazil has lost over 615,636 lives due to COVID-19 since the beginning of the pandemic, and presently, the ICU admission level is at its lowest level since March 2020. Among the pediatric and young-adult population (<20 years of age) who were infected with COVID-19 and admitted to the hospital due to severe acute respiratory disease (SARS), 86.5% were discharged, 7.6% died, 3.2% were still in the hospital when this analysis was being conducted, and 2.7% had no reported outcome. Of this population, the risk of mortality increased in infants less than two years old, adolescents of ages 12-19, children in indigenous, northeastern, and northern regions, and children with pre-existing diseases. The most common comorbidities were asthma and neurological disorders, yet surprisingly, kidney disease was only present in 1.5% of patients. 65.8% of the population have received 2 doses of vaccine and 75% one dose Disease control is associated with vaccination, which although started later than in other countries was efficiently administered. The population has recognized the importance and success of vaccination.

Dr. Tavares and Dr. Koch point to a significant issue arising due to the unexpected financial burden in Brazil. The pandemic costs in Brazil were high and unexpected. The 2022 Brazilian federal healthcare budget will include continuous financing of COVID-19 prevention and care which will result in significantly diminished investments for the other areas of care, decreasing the quality and quantity of health care in the future. Therefore, due to the negative impact forced upon the public health system, pediatric peritoneal dialysis may be shut down and result in less favored patients being treated with hemodialysis. Although the Brazilian representatives raised this issue, one should expect similar financial burdens in many other countries.

## COVID-19 in India

Dr. Bagga’s presentation reinforces the detrimental impact COVID-19 has on the kidney and describes the overarching practice of nephrology in India. Dr. Bagga reported India having the second-highest cases globally, as of August 2021. This consists of approximately 50,000 cases/day and 500 deaths/day. India has been the victim of two pandemic waves, of which the second one demonstrated more severe complications, including 4 times more cases of MIS-C. In India, 10-12% of children have shown severe complications, including AKI, due to COVID-19. In a study focused on AKI in children with COVID-19, Dr. Bagga presented a patient population of 295 positives for SARS-CoV2 in which 105 patients were admitted and 24 (22.8%) of those patients were diagnosed with AKI. Risk factors such as sepsis, nephrotic syndrome, vasopressor support, and mechanical ventilation were accounted for. He reported that 27 (26%) patients died. Additionally, in a retrospective study discussing COVID-19 in children with CKD, Dr. Bagga described a population of 88 patients with a median age of 10. Of the 25 patients (34%) who were diagnosed with AKI, 12 had AKI stages 1-2, 11 suffered respiratory failure, 4 suffered from shock, 1 suffered from encephalopathy, and 3 died. Lastly, Dr. Bagga stressed the importance of factors such as patients, caregivers, and healthcare personnel when managing children with renal disease during COVID-19.

## Policies and regulations: Singapore, UK, South Africa

Given the severity of the COVID-19 outbreak, healthcare systems across the globe were detrimentally impacted. While populations were vulnerable, several countries such as Singapore, the UK, and South Africa shared their insight on how they continue to effectively combat the pandemic and strengthen their strategies for future global health challenges.

Representing Singapore, Dr. Hui-Kim Yap, professor in the Department of Pediatrics at Yong Loo Lin School of the Medicine National University of Singapore, discussed the COVID-19 pandemic and its impact on children’s dialysis programs in Singapore. Previously in March of 2003, Singapore experienced one of the most significant outbreaks of the severe acute respiratory syndrome. The novel coronavirus emerged in January 2020, with over 65,000 confirmed cases, nearly 2,000 active cases, 63,000 estimated recovered cases, 41 deaths, and a fatality rate of 0.06%. With COVID-19’s growing prevalence, Dr. Yap described the early mask mandate for the dialysis team at her center for chronic dialysis programs for children. A split team policy was implemented in which the center was divided into two, and distinguished groups were refrained from encountering one another unless wearing an N-95 mask.

Furthermore, dialysis patients with fever or upper respiratory infection (URTI) symptoms were separated from asymptomatic patients who had been near COVID patients. Postponing kidney transplant surgeries posed a challenge as the number of dialysis patients increased but limited nursing personnel. This resulted in planning preemptive transplant patients requiring dialysis but simultaneously adhering to protocols to avoid COVID-19 infections in the dialysis unit. Dr. Yap emphasized how Singapore demonstrated effective and efficient policymaking to prioritize the utmost safety of its patients and staff. Currently, over 70% of Singapore’s population is fully vaccinated–demonstrating successful policymaking.

Dr. McCulloch, PICU Consultant and Paediatric Nephrologist at Red Cross War Memorial Children’s Hospital represented South Africa, explained the prevalence of COVID-19 among pediatric populations. With the 17th highest COVID rate globally, South Africa has endured almost 2.5 million cases and three waves. Despite the minuscule child COVID mortality rate, this number continues to increase as the Delta variant spreads. Like the UK, they also faced the obstacle of communication, Dr. McCulloch discussed how, unfortunately, inadequate communication could belittle specific populations and care should be taken to avoid stigmas within the hospital setting of COVID, which could lead to the rampant spread of the virus and mistrust of medical professionals.

## Delta variant: South Africa, the UK

According to the Center for Disease Control (CDC), the Delta variant is believed to be twice as more contagious than previously reported variants and may lead to severe illness in unvaccinated individuals. Vaccination is of utmost importance when considering preventative measures to combat the Delta variant. Though vaccination does not guarantee complete protection against the variant, it reduces the risk of severe sickness and death. However, fully vaccinated individuals with the Delta variant may still be carriers. In addition, mask-wearing in indoor settings and public places reduces the transmission of the variant.

Dr. Mok mentions how case numbers are vastly increasing due to the Delta variant. Similarly, Dr. McCulloch described how the Delta variant is 1000x more contagious than the beta variant experienced prior. Furthermore, she suggested that more children present with the Delta variant even though these children have lower hospitalization rates. The efficacy of the J&J vaccine was also discussed based on two data sets; the J&J vaccine demonstrated 65-66% protection against hospitalization and 91-95% protection against death. While there is speculation surrounding which vaccines are better prepared to resist the Delta variant, knowledge is scarce, and few studies are underway.

Addendum: After this congress, Omicron became the major COVID-19 variant worldwide with much higher uptake in pediatric cases but still significantly fewer critical cases needing intensive care admissions in children compared to unvaccinated adults.

## Moving forward: renal complications summary, policy recommendations, and current research

The virtual international conference, Pediatric Critical Care Nephrology & Dialysis Course, discussed critical research breakthroughs on renal complications associated with COVID-19 in addition to recommended treatments and techniques. AKI is an adverse prognostic factor of COVID-19 in pediatric populations. While KRT has been associated with lower mortality rates in children with AKI, research continues to show complications, such as progression to severe stages of the disease, high mortality, and increased length of hospital stay. Additionally, a causal relationship between CKD and COVID-19 in pediatric populations has not yet been established, however, it is evident that renal complications contribute to significant health ramifications. For example, research shows a high prevalence of MIS-C in pediatric populations. It is integral to observe cardiac function (heart rate and blood pressure) and kidney function (fluid status and urine output) to execute beneficial treatment plans. Furthermore, the research investigated the clinical course of COVID-19 in immunosuppressive patients and indicated that the monoclonal antibody medication, Rituximab, does not increase the risk of COVID-19 infection in pediatric patients nor does its use differentiate from general pediatric populations. For pediatric patients on immunosuppressive therapy, it is recommended that children follow similar masking and social distancing measures as their healthy counterparts. Concerns surrounding vaccine administration in renal transplant patients suggested that vaccination may potentially interfere with sufficient antibody levels and contribute to transplant rejection.

Implementing mask mandates, distinguishing physician groups, and planning preemptive transplant patients were useful strategies in reducing the risk of COVID-19. Additionally, physicians and policymakers should function as a cohesive unit to ensure that all citizens are adhering to the preventative measures being put forth. Masking, at the very least, and vaccination programs should be mandated and accessible, especially to nations that face significant financial burden due to the pandemic and are low-income. This will help avoid excessive hospitalizations and deaths. Considering all global perspectives, it is evident that prioritizing organization, communication, and utmost safety of patients and medical personnel through masking and vaccination will ultimately combat future health challenges in an effective and efficient manner. **Tables 5**, **6** outline potential next steps forphysicians, policymakers, and research as well as critical takeaways of theconference.

**Table 5:** Comprehensive list of next steps for physicians, policymakers, and research.

• Physicians and policy workers should function as a cohesive unit to ensure that all citizens are adhering to preventative measures being put forth. • Mask mandates should be strictly upheld. • Vaccination programs should be readily accessible and strictly upheld, with emphasis on low-income countries or nations facing significant financial burdens due to the pandemic. • Further research must analyze the evolution of the virus, with factors such as variants, to better understand the morbidity and mortality associated with the virus.

**Table 6:** Conference summary.

• The Pediatric Critical Care Nephrology & Dialysis on August 7th, 2021 provides international perspectives on COVID-19 in connection to pediatric nephrology • COVID-19 complications are extending beyond respiratory distress to other, more widespread complications, such as AKI or MIS-C, even in pediatric patients. • This conference suggests that children on immunosuppressive therapy have no current necessity to isolate to a greater extent, and hence should follow the same protection measures as other children. • This conference raises concerns about vaccine safety in renal transplant patients, as well as reduced vaccine efficacy in organ transplant recipients • Various professionals describe the COVID-19 pandemic in Britain, Latin America, India, Singapore, UK, and South Africa. These discussions described the current pandemic conditions, and policies and regulations pertaining to the outbreak. • This conference describes the Delta variant as contagious and severe. The Omicron variant is described as contagious and of lower severity.

Although there still continues to be gaps within our knowledge on the deleterious effects of COVID-19 on the development of renal complications in pediatric populations, a retrospective analysis conducted by Raina et al. demonstrated that out of 2,546 COVID+ pediatric patients, 10.8% (n=274) were diagnosed with AKI (7). Factors such as length of hospital stay, mortality rate, respiratory support, and kidney support were investigated and showed significantly higher categorical outcomes. Though statistics show milder effects of AKI on the pediatric COVID+ population, it is still worth recognizing that this relationship exists between COVID and renal complications and further research is needed to understand how to tackle this prevalent health issue.

## Data availability statement

The original contributions presented in the study are included in the article/supplementary materials. Further inquiries can be directed to the corresponding authors.

## Author contributions

Investigation: NV and RK. Resources and presentation: VK, HY, KA, GM, MM, MB-F, AB, QM, MST, FS, CF, and TB. Original draft preparation: NV and RK. Review and editing: NV, RK, RR, NN, TN, and SS. Supervision: RR, NN, and SS. Project administration: RR. All authors contributed to the article and approved the submitted version.

## Conflict of interest

The authors declare that the research was conducted in the absence of any commercial or financial relationships that could be construed as a potential conflict of interest.

## Publisher’s note

All claims expressed in this article are solely those of the authors and do not necessarily represent those of their affiliated organizations, or those of the publisher, the editors and the reviewers. Any product that may be evaluated in this article, or claim that may be made by its manufacturer, is not guaranteed or endorsed by the publisher.
