# Supplementary material for: PCRRT-ICONIC critical care pediatric nephrology course: the global prevalence of COVID-19 and associated sequelae
Source: Front Nephrol. 2022 Oct 5;2:1008629. doi: 10.3389/fneph.2022.1008629 (PMC10479684; doi:10.3389/fneph.2022.1008629)
Supplement: Supplementary file 1 [file Table_1.docx]

**Supplemental Table 1.** 2020 Global Survey, Dr. Schaefer

| Sample Population | n=113 COVID-19 patients, 0-20 years |
| --- | --- |
| Kidney transplantation | 46% |
| Nephrotic syndrome | 26% |
| SLE | 10% |
| ANCA Vasculitis | 2% |
| IgA nephropathy | 2% |
| aHUS | 2% |
| Other glomerulonephritis | 6% |
| Other complications | 4% |

**Supplemental Table 2.** Multi-organ system involvement in COVID-19 infection, Britain

| Renal Involvement | 91% |
| --- | --- |
| Neuronal Complications | 50% |
| Gastrointestinal Involvement | 98% |
| Prothromtic States | 87% |
| Echo abnormalities/Increased troponin/NT-proBNP | 33% |

**Supplemental Table 3.** Comprehensive list of next steps for physicians, policymakers, and research

| - Physicians and policy workers should function as a cohesive unit to ensure that all citizens are adhering to preventative measures being put forth. - Mask mandates should be strictly upheld. - Vaccination programs should be readily accessible and strictly upheld, with emphasis on low-income countries or nations facing significant financial burdens due to the pandemic. - Further research must analyze the evolution of the virus, with factors such as variants, to better understand the morbidity and mortality associated with the virus. |
| --- |
